# Supplementary material for: Ultrasonic-Assisted Extraction of Phenolic Compounds from Lonicera similis Flowers at Three Harvest Periods: Comparison of Composition, Characterization, and Antioxidant Activity
Source: Molecules. 2024 Jul 11;29(14):3280. doi: 10.3390/molecules29143280 (PMC11279271; doi:10.3390/molecules29143280)
Supplement: Supplementary file 1 [file molecules-29-03280-s001.zip › molecules-3058053-supplementary.pdf]

Table S1. BBD using different combinations of factors and their levels for LSF-PC.

Numbers	Factors				Response			
	A:Ultrasound power (W)	B: Ethanol concentration (%)	C: SLR	D:Extraction Time (min)	TPC content (mg GAE/g)		TFC content(mg RE/g)	
					Actual Value	Predicted Value	Actual Value	Predicted Value
1	-1(100)	-1(20)	0(30)	0(15)	96.36±2.30	97.05	55.79±0.48	59.41
2	-1(100)	0(50)	-1(15)	0(15)	110.13±1.98	105.17	60.58±0.62	59.45
3	-1(100)	0(50)	0(30)	-1(5)	94.59±1.32	97.81	56.68±0.97	57.23
4	-1(100)	0(50)	0(30)	1(25)	111.36±0.47	113.03	69.22±1.82	66.07
5	-1(100)	0(50)	1(45)	0(15)	101.39±1.77	100.60	67.52±2.87	65.70
6	-1(100)	1(80)	0(30)	0(15)	86.44±2.30	86.61	50.17±2.67	52.10
7	0(300)	-1(20)	-1(15)	0(15)	97.28±1.60	100.52	53.73±2.82	51.31
8	0(300)	-1(20)	0(30)	-1(5)	97.28±1.07	95.33	55.46±1.22	54.06
9	0(300)	-1(20)	0(30)	1(25)	102.75±0.95	100.79	52.74±0.77	54.36
10	0(300)	-1(20)	1(45)	0(15)	91.12±2.28	90.53	59.59±1.12	58.97
11	0(300)	0(50)	-1(15)	-1(5)	106.59±0.57	106.37	54.44±3.18	55.21
12	0(300)	0(50)	-1(15)	1(25)	110.59±2.10	111.92	54.91±2.84	56.38
13	0(300)	0(50)	0(30)	0(15)	116.90±3.13	114.90	62.03±3.18	65.38
14	0(300)	0(50)	0(30)	0(15)	113.05±3.20	114.90	67.46±1.41	65.38
15	0(300)	0(50)	0(30)	0(15)	114.75±1.71	114.90	66.64±1.7	65.38
16	0(300)	0(50)	1(45)	-1(5)	99.20±3.63	98.61	59.49±0.66	59.16
17	0(300)	0(50)	1(45)	1(25)	102.31±1.84	103.28	64.78±0.72	65.15
18	0(300)	1(80)	-1(15)	0(15)	90.36±3.23	90.60	49.42±1.29	48.50
19	0(300)	1(80)	0(30)	-1(5)	85.98±3.54	87.54	47.86±1.33	46.66
20	0(300)	1(80)	0(30)	1(25)	90.75±0.89	92.31	51.73±0.35	53.53
21	0(300)	1(80)	1(45)	0(15)	87.77±1.39	84.19	52.67±3.35	53.54
22	1(500)	-1(20)	0(30)	0(15)	93.82±3.38	94.39	49.08±2.52	48.29
23	1(500)	0(50)	-1(15)	0(15)	108.05±3.49	108.45	49.19±2.67	51.42
24	1(500)	0(50)	0(30)	-1(5)	109.59±2.64	107.57	52.95±3.07	54.55
25	1(500)	0(50)	0(30)	1(25)	106.13±1.88	102.56	54.98±2.37	52.88
26	1(500)	0(50)	1(45)	0(15)	92.04±2.21	96.62	56.34±2.44	57.87
27	1(500)	1(80)	0(30)	0(15)	88.52±3.12	88.57	49.83±2.99	47.36

Table S2 Mobile phase parameters and standard curves of nine phenolic compounds

Substance	Chemical formula	Mobile phase A	Mobile phase B	Time and proportion	Standard curve	R^2
Rutin	$C_{27}H_{30}O_{16}$	water-phosphoric acid (1%)	Methanol	0-8min 30%, 15-25min 65%	y = 3.6195 x + 0.0640	0.9999
Quercetin	$C_{15}H_{10}O_7$	water-phosphoric acid (1%)	Methanol	0-8min 30%, 15-25min 65%	y = 7.1294 x - 0.1801	0.9999
Luteolin	$C_{15}H_{10}O_6$	water-phosphoric acid (1%)	Methanol	0-8min 30%, 15-25min 65%	y = 10.4518 x - 0.9092	0.9998
Kaempferol	$C_{15}H_{10}O_6$	water-phosphoric acid (1%)	Methanol	0-8min 30%, 15-25min 65%	y = 8.8767 x - 0.3402	0.9999
Epicatechin	$C_{15}H_{14}O_6$	water-phosphoric acid (1%)	Methanol	0 min 10%, 22 min 43%	y = 5.7116 x - 0.0810	0.9999
Caffeic acid	$C_9H_8O_4$	water-phosphoric acid (1%)	Methanol	0 min 40%	y = 23.7543 x - 1.0191	0.9998
Ferulic acid	$C_{10}H_{10}O_4$	water-phosphoric acid (1%)	Methanol	0-10 min 20%, 20-25 min 60%	y = 36.4180 x - 0.6987	0.9999
Isoquercitrin	$C_{21}H_{20}O_{12}$	water-phosphoric acid (1%)	Acetonitrile	0 min 20%	y = 11.1103 x - 4.0684	0.9999
Chlorogenic acid	$C_{16}H_{18}O_9$	water-phosphoric acid (1%)	Methanol	0-10min 20%, 20-25min 60%	y = 16.2829 x - 4.6999	0.9999
